# Assembly of Semiconductor Nanorods into Circular Arrangements Mediated by Block Copolymer Micelles

**DOI:** 10.3390/ma15082949

**Published:** 2022-04-18

**Authors:** Riham Muzaffar-Kawasma, Meirav Oded, Roy Shenhar

**Affiliations:** Institute of Chemistry and the Center for Nanoscience and Nanotechnology, The Hebrew University of Jerusalem, Jerusalem 9190401, Israel; riham.kawasma@mail.huji.ac.il (R.M.-K.); meirav.oded@mail.huji.ac.il (M.O.)

**Keywords:** self-assembly, block copolymer micelles, nanorods, quantum rods, nanoparticle superstructures

## Abstract

The collective properties of ordered ensembles of anisotropically shaped nanoparticles depend on the morphology of organization. Here, we describe the utilization of block copolymer micelles to bias the natural packing tendency of semiconductor nanorods and organize them into circularly arranged superstructures. These structures are formed as a result of competition between the segregation tendency of the nanorods in solution and in the polymer melt; when the nanorods are highly compatible with the solvent but prefer to segregate in the melt to the core-forming block, they migrate during annealing toward the core–corona interface, and their superstructure is, thus, templated by the shape of the micelle. The nanorods, in turn, exhibit surfactant-like behavior and protect the micelles from coalescence during annealing. Lastly, the influence of the attributes of the micelles on nanorod organization is also studied. The circular nanorod arrangements and the insights gained in this study add to a growing list of possibilities for organizing metal and semiconductor nanorods that can be achieved using rational design.

## 1. Introduction

In nanotechnology, a key issue for the fabrication of functional devices and nanostructures is the controlled assembly of functional nanoparticles (NPs) into ordered arrangements that have a specific set of properties serving the desired application [1,2,3]. The assembly of rod-shaped NPs, or nanorods (NRs), is of a particular interest because their shape anisotropy gives rise to different packing modes (e.g., end-to-end and side-to-side), which, in turn, strongly influence the collective properties of the ensemble [2,4,5,6,7,8,9,10,11,12]. For example, the longitudinal and transverse plasmon resonances of gold NRs, which are affected by NR–NR interaction, can be finely tuned by controlling their packing mode [12,13,14].

Expanding the assembly options from the structures offered by the natural packing tendency of the NRs to more elaborate, predefined structures requires the utilization of a guiding mechanism [14,15]. Modification of substrates by top-down lithographic means provides a popular way for the integration of a guiding mechanism [16]. Yet, for many applications that require ordering in the sub-micrometer regime, lithography becomes exceedingly expensive (because photolithography is irrelevant at this length scale), whereas direct-write techniques [17] do not necessarily deliver the line edge smoothness that is required for assembling nanoscale objects. Additionally, some of the benefits provided by lithography, such as the high precision in the placement of features, are often unnecessary.

A conceptually different approach to bias the natural assembly tendency of NPs relies on the incorporation of another component, which serves as a template onto which NPs can assemble. Thus, the NP ensemble inherits the structural characteristics of the added component. For example, self-assembled DNA strands were used to template the organization of plasmonic NPs into precise circular arrays, which exhibited shape-dependent plasmon resonance modes [18]. If the added component additionally tends to self-organize into ordered arrays (e.g., latex spheres), its utilization for organizing NPs will result in arrays of NPs exhibiting morphologies that would be influenced not only by the structure or shape of the added component but also by its mode of assembly. Hence, successful application of this bottom-up approach requires knowledge of the way NPs (spherical, rod-shaped, or others) and structural components interact and the principles that govern the assembly of the structural components.

Block copolymers (BCPs) provide perhaps the most versatile self-structuring materials for submicron and nanoscale fabrication. These polymers consist of sequences (blocks) of chemically distinct monomers; when these blocks are also chemically incompatible, they naturally form nanoscale structures by phase separation [19]. In thin films, BCPs exhibit periodic structures of controlled morphology and periodicity, which could serve as templates for organizing NRs [20,21]. Russell and coworkers selectively assembled CdSe NRs in grooves made in polystyrene-*block*-poly(methyl methacrylate) (PS-*b*-PMMA) films [22]. Plawsky et al. applied the same approach to Au NRs [23], and Sohn et al. demonstrated dichroism in oriented Au NR arrays using BCP films assembled on topographically patterned substrates [14]. Our group demonstrated hierarchical structures of CdSe NRs on the film surface organized in smectic-like arrangements, which were obtained by cooperative assembly [9,10].

Another mode by which BCPs can serve as bottom-up templates for NP organization, which has been rarely investigated so far, relies on the propensity of amphiphilic BCPs to form micelles when placed in a selective solvent, where the insoluble blocks form the core, and the soluble blocks form the corona. The morphology of the micelles formed above the critical micelle concentration (CMC) can be spherical, cylindrical, or vesicular depending on the packing parameter of the BCP chains, which is influenced by the attributes of the BCP blocks (i.e., the volume occupied by the core-forming chains, the interfacial area, and the maximum length of the insoluble chains). Asymmetric diblock copolymers can give rise to either hairy (starlike) micelles or crew-cut micelles depending on the relative length of the corona-forming block (long or short, respectively) [24,25,26,27,28].

Block copolymer micelles have been mostly utilized as nanoreactors for NP synthesis inside their cores (pioneered by Möller and Spatz) [29,30,31,32,33,34,35,36,37,38,39,40]. Compared to this body of work, the employment of BCP micelles as predefined templates for mediating NP assembly has been less investigated [41,42,43,44,45,46,47,48,49]. Winnik et al. used polystyrene-*block*-poly(2-vinyl pyridine) (PS-*b*-P2VP) crew-cut micelles to assemble semiconductor quantum dots (QDs) at the core–corona interface and a conjugated polymer inside the cores to mimic light-harvesting systems [43]. Kim and coworkers designed multicolor-emitting BCP/QDs microspheres by encapsulating QDs inside the cores of PS-*b*-P2VP micelles [45]. In another study by Jiang and Nie, the controlled co-assembly of PS-coated QDs inside polystyrene-*block*-poly(ethylene oxide) micelle cores gave rise to diverse QD/BCP morphologies featuring high luminosity and water solubility [47].

Recently, Rafipoor et al. used micelles to cluster semiconductor quantum rods (QRs) in their cores and demonstrated their potential as biomedical imaging probes [48]. Sohn et al. organized gold nanorods modified with PS ligands into hexagonal networks using close-packed, hairy PS-*b*-P4VP micelles. The chemical compatibility between the PS-modified Au NRs and the matrix formed by the micelle coronas guided the NRs to organize around the micelles with their long axes oriented tangentially to the perimeter of the micelles [50]. The authors presented the aspect of organizing one-dimensional NRs within zero-dimensional micelles, and they showed that co-assembly (i.e., the formation of ordered arrays with hexagonal NR arrangement) was only possible when the micelle diameter was larger than the NR length.

In the current study, we demonstrate the formation of circular arrangements of semiconductor QRs around BCP micelles. Insights gained into the self-assembly process elucidate the role of the balance across the interactions of the QRs with the different blocks and with the solvent, the mutual influence between the QRs and the BCP micelles, and the influence of the micelle structure on the assembly results.

## 2. Materials and Methods

*Materials.* Polystyrene-*block*-poly(2-vinyl pyridine) copolymer (PS-*b*-P2VP; *M*_n_ 270 kDa, PDI 1.23, *f*_PS_ = 0.35; denoted as PS_89_-*b*-P2VP_181_, where the subscript number denotes the *M*_n_ values of the respective blocks) was synthesized by standard anionic polymerization using *sec*-butyllithium in THF under nitrogen atmosphere. The molecular weight, size distribution, and polystyrene weight fraction were all determined by gel permeation chromatography (GPC) in tetrahydrofuran (THF) against PS standards for the PS block and comparison of the ^1^H-NMR signals for phenyl and pyridine groups for the P2VP block. Other PS-*b*-P2VP copolymers (*M*_n_ 199 kDa, PDI 1.12, *f*_PS_ = 0.53 and *M*_n_ 536 kDa, PDI 1.23, *f*_PS_ = 0.73; denoted as PS_102_-*b*-P2VP_97_ and PS_380_-*b*-P2VP_156_, respectively) were purchased from Polymer Source, Inc., and were used as received. CdSe-seeded CdS quantum rods coated with trioctylphosphine oxide (TOPO) ligands were received in crude form (batch number 15KL022) from Qlight Nanotech (Jerusalem, Israel; a Merck subsidiary), dissolved in toluene, and purified from excess TOPO by two cycles of centrifugation (3000 rpm for 10 min, and 2000 rpm for 8 min at 15–20 °C) using methanol as an anti-solvent. The QRs measured 24 ± 2 nm in length and 6.3 ± 1 nm in diameter, as determined from SEM images of self-assembled QRs on a SiOx substrate using ImageJ software (version 1.52v, W. S. Rasband, U.S. National Institutes of Health, Bethesda, MD, USA, 1997–2020, http://imagej.nih.gov/ij/) [51], and the grafting density was ca. 2.1 ligands·nm^−2^ (~1000 ligands/QR, calculated from thermogravimetry analysis determination of 16.3 wt.% bound TOPO ligands).

*Preparation of micellar films.* Stock solutions of the copolymers in toluene (2–3.5 wt.%) were stirred for 2 h at room temperature and left to equilibrate overnight before use. QRs were also dispersed in toluene at different concentrations. A stock solution of QRs in toluene was prepared dissolving ~800 mg of purified QRs in 3 mL toluene, resulting in ~7 μM concentration (determined using UV/Vis spectroscopy, with $\varepsilon_{350}$ = 2.12 × 10^7^ M^−1^cm^−1^) [52]. The BCP solutions were combined with different volumes of the QR solution (typically in the range of 0–100 μL for a total nanocomposite solution of 700 μL), resulting in solutions featuring final BCP concentrations of 0.5, 1.0, and 1.5 wt.% and different QR/BCP ratios (actual filling fractions were eventually determined directly from the SEM images, see below). The combined solutions were left to equilibrate overnight in closed vials and filtered using 0.22 μm PTFE syringe filters. The filtered nanocomposite solutions were spin-coated at 3000 rpm for 30 s onto clean silicon substrates (covered with ca. 2 nm native oxide). The silicon wafers were cleaned using NoChromix acid bath (Godax Laboratories, Bethesda, MD, USA). Each sample was cut into four pieces for separate use. The films were annealed in closed Petri dishes under saturated atmospheres of toluene or chloroform vapors for 15 min at room temperature, after which they were quenched by opening the lid and quickly removing the samples.

*Characterization.* Scanning electron microscopy (SEM) images were acquired using an extra-high-resolution scanning electron microscope Magellan 400L (Thermo Fisher Scientific, former FEI, Hillsboro, OR, USA) operating at 2 kV and 0.1 nA. Tilted images (35–45°) were acquired at either 2 or 5 kV.

QR filling fractions in the nanocomposite films were quantified as the fractional area occupied by the QRs in the film (termed as “surface fraction” hereafter), which was calculated by counting QRs in a 0.5–1.5 μm^2^ area (which included a few hundred QRs) and using 152 nm^2^ as the approximate area occupied by a single QR (determined from its dimensions as described above). The surface fraction remained constant within experimental error after annealing. Additionally, to elucidate the change in QR location upon annealing, the QRs in relevant systems were counted and classified into four categories: QRs located at the core periphery (i.e., core–corona interface), QRs belonging to an aggregate, isolated QRs located in the region between micelles, and QRs appearing on top of the micelles. The number fractions of QRs of each category were determined (out of 1000–2400 counted QRs for each system); the percentage values reported in Supplementary Figure S1 represent average values and standard deviations determined from images taken at three different areas on each of the corresponding samples.

Atomic force microscopy (AFM) images for film topography and height profiles were obtained in tapping mode using a Dimension Icon XR Scanning Probe Microscope (Bruker Corporation, Billerica, MA, USA). For height analysis, depth histograms from AFM images acquired near a scratch in the film were generated by the Nanoscope Analysis 2.0 software (Supplementary Figure S2a,c,e). The borderline of the scratch was masked to avoid the influence of accumulated material on the depth analysis. Depth histograms were deconvoluted into 3–5 Gaussians. The Gaussian corresponding to the deepest features represented the scratch and was, thus, assigned zero height (in some samples, two Gaussians were required to fit the data of the scratch because of polymer residues remaining in it). The Gaussian corresponding to the highest features was associated with the micelle cores; the remaining Gaussians were attributed to the corona and QR-decorated corona blocks. Diameter histograms of the micelles were obtained from SEM images by fitting ovals to the shapes of the micelle cores using ImageJ software [51]; the calculated areas were used to determine the effective diameters (i.e., of circles with the same areas; Supplementary Figure S2b,d,f).

## 3. Results and Discussion

**Figure 1:**
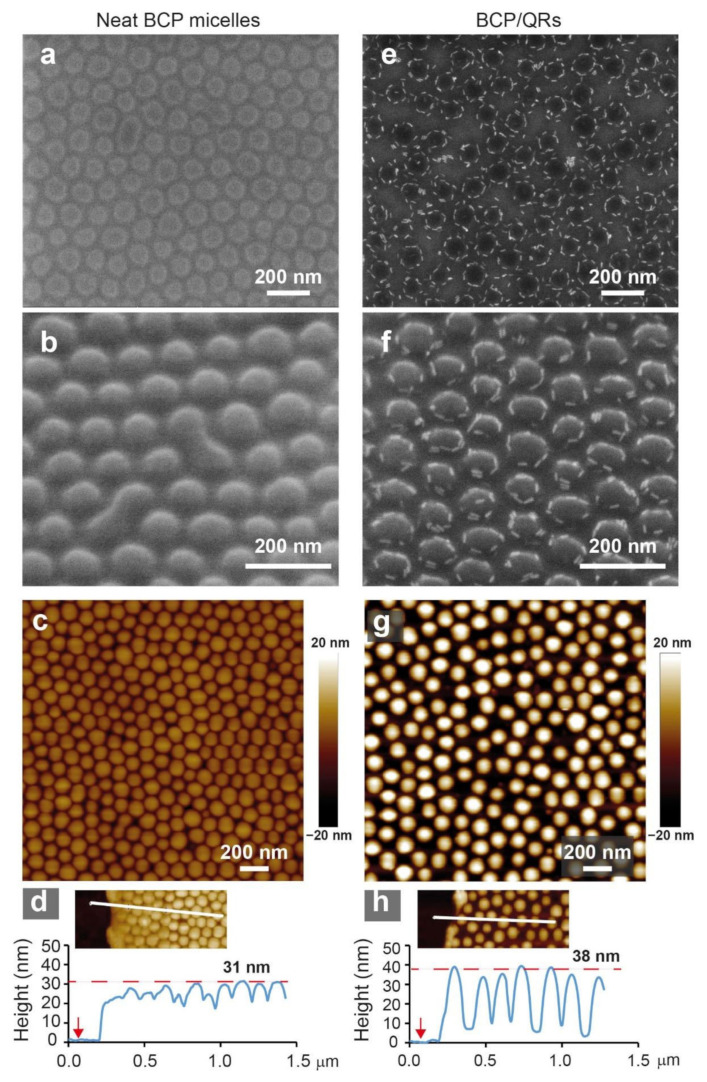
Top view (**a**,**e**) and tilted (**b**,**f**) SEM images and AFM images (**c**,**g**) of micellar PS_89_-*b*-P2VP_181_ films cast from 0.5 wt.% toluene solutions and annealed in toluene vapor. (**a**–**c**) Neat BCP films. (**e**–**g**) BCP films containing CdS QRs (surface fraction σ_QR_ = 0.08). (**d**,**h**) AFM height cross-sections made near a scratch in the film corresponding to the images shown in panels (**c**,**g**); red arrows denote the bare substrate level.

Casting a 0.5 wt.% PS_89_-*b*-P2VP_181_ solution in toluene on a SiOx substrate followed by 15 min annealing in the vapor of the same solvent at ambient temperature gave rise to a stable array of flattened micelles, with an average diameter of 89 ± 9 nm and average height of 31 ± 2 nm (Figure 1a–c and Figure S2c,d). The dimensions of the flattened micelles depended on their density; casting a 0.3 wt.% solution led to structures that were wider (102 ± 10 nm in diameter) and flatter (22 ± 3 nm in core height; Supplementary Figure S2a,b), suggesting that the lower areal density of deposited micelles enabled them to spread more on the substrate.

Interestingly, when TOPO-coated CdS QRs were added to the micellar BCP solution cast from a 0.5 wt.% solution, the cast and annealed films revealed the formation of an array of micelles decorated by QRs at the periphery of the P2VP cores (Figure 1e–g). Observing these structures at a tilt (Figure 1f) showed that the QRs adhered to the core–corona interface of the micelles. The QR-decorated micelles appeared less flattened than the neat micelles; comparing Figure 1c,d to Figure 1g,h and a more extensive statistical analysis (Supplementary Figure S2e,f; see Section 2 for further details) revealed that their cores are extended in the direction normal to the substrate (37 ± 4 nm core height) and shrunk in the lateral dimensions compared to the neat micelles (85 ± 8 nm core diameter). The statistical height analysis (Supplementary Figure S2e) also revealed two height values for the corona regions in isolated micelles, attributed to the height of the spread corona blocks (8 ± 1 nm) and the total height of corona and QR (11 ± 2 nm).

The structures shown in Figure 1e–g are not trivial in two aspects. First, TOPO-coated QRs are known to be P2VP-compatible owing to the metal–ligand interaction between the Cd surface atoms and the pyridine groups [43,53,54]. Indeed, blending QRs with PS homopolymer leads to phase separation and QR aggregation, whereas blending QRs with P2VP homopolymer leads to a homogeneous dispersion (see Supplementary Figure S3), attesting to the difference in compatibility of the QRs with the different polymers. Hence, one might expect that the BCP micelles incorporate the QRs in their cores. Apparently, the PS blocks forming the corona of the micelles create a barrier for penetration of the QRs into the micelle cores while in solution; consequently, upon casting, the QRs end up in the PS regions (Using QRs that were more gently purified from the excess ligand (i.e., with only one centrifugation cycle) led to more random adsorption to the micelles’ surfaces upon casting (Supplementary Figure S4). This behavior suggests that the excess ligand made these QRs actually compatible with the PS coronas, which led these QRs to interact with the PS coronas already in solution instead of being preferably dispersed in the solvent. Apparently, incompatibility of the QRs with the PS coronas is essential for obtaining the circular arrangement.) Second, nanoparticles in nanocomposite films usually tend to segregate to the interfaces of the film (both film/substrate and the free surface) to lower the interfacial tension of the film [10,11,42,55,56]. However, in the micellar nanocomposite film, the QRs surrounded the periphery of the micelles without covering their top surface. This means that the QR assembly took place in a quasi-two-dimensional fashion. Taken together, it must be concluded that the resulting structures represent a kinetically trapped, nonequilibrium state that is accessed by the choice of casting and annealing conditions. Nonetheless, this nonequilibrium state is reproducibly obtained.

**Figure 2:**
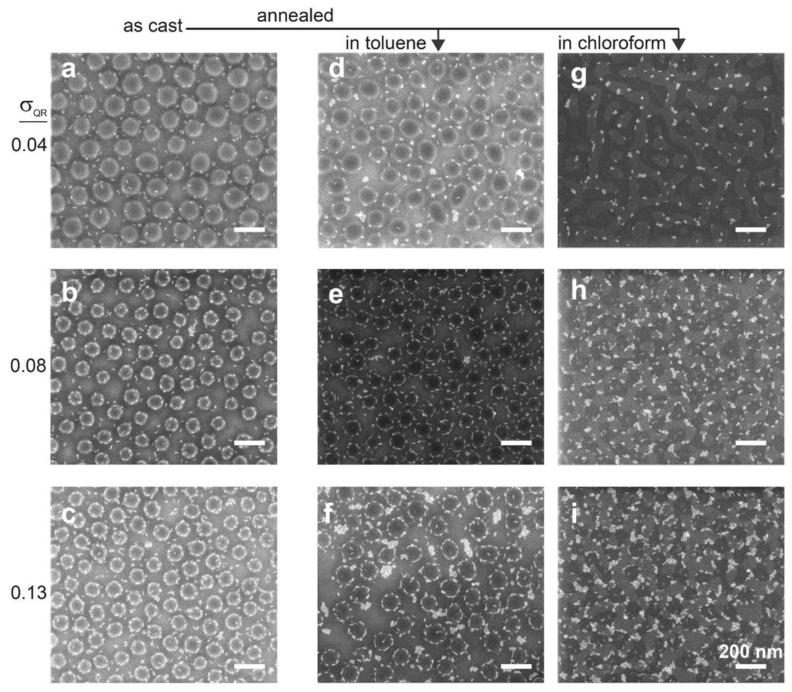
Effect of annealing solvent and QR filling fraction in 0.5 wt.% BCP-based nanocomposites cast from toluene solutions: (**a**–**c**) as-cast films; (**d**–**f**) films annealed in toluene vapor; (**g**–**i**) films annealed in chloroform vapor. The QR filling fractions are quantified as observed surface fraction (σ_QR_); see Section 2 for additional details.

Aiming to reveal the factors governing the formation of the QR-decorated micelle structure, we inspected samples before and after annealing and compared two annealing solvents. Figure 2 shows SEM images of nanocomposite films, both as-cast and annealed under saturated toluene or chloroform vapor. Images taken of as-cast films at the lowest filling fraction (σ_QR_ = 0.04, where σ_QR_ represents the observed surface fraction) showed that the majority of the QRs were located in the regions between deposited micelles, whereas some were already adsorbed to the micelle periphery (Figure 2a). Annealing in toluene vapor increased the fraction of QRs located at the micelle periphery from 34% to 55% (Figure 2d; see Supplementary Figure S1). Additionally, most of the QRs that were still located in the region between the micelles appeared aggregated, and only a few isolated QRs were observed. These observations suggest that, in solution, the QRs were not co-assembled with the BCP micelles. This can be explained by their high solubility in toluene, which is also selective to the PS blocks. When the film was cast, these conditions caused a large fraction of the QRs to be initially located within the PS domains in the film, as concluded above. The incompatibility between the QRs and the PS domains created a driving force for lateral QR migration toward the P2VP domains and their adhesion to the core–corona interface. This migration, which apparently started already as the film was drying, continued during solvent vapor annealing and eventually led to the “crowned” appearance. The confinement within the ~10 nm film thickness (the average thickness of the PS corona in the flattened adsorbed micelles; see Supplementary Figure S2) inhibited the ability of the QRs to migrate to the top of the micelle. Nonetheless, after reaching the PS/P2VP interface, the QRs did not continue and penetrate the P2VP cores. This suggests that their location at the periphery of the micelle core lowered the interfacial tension between the blocks.

Increasing the QR filling fraction to σ_QR_ = 0.08 also increased the fraction of QRs located at the micelle periphery already in the as-cast films to 68% (Figure 2b and Figure S1). This observation suggests that, immediately upon casting a medium filling fraction sample, a larger fraction of QRs ended up located at the vicinity of micelles; these QRs were, thus, strongly attracted to the nearest micelles and migrated toward them as the film dried. Annealing in toluene vapor increased the fraction of QRs located at the periphery of micelles to 78% (Figure 2e and Figure S1), indicating that annealing also allowed QRs located a little farther away from the nearest micelles to reach their periphery. An additional increase in filling fraction to σ_QR_ = 0.13 resulted in a reduction in the filling fraction of QRs at the core–corona interface in the as-cast film to 65%, which did not change much after annealing (Figure 2c,f and Figure S1). The reason for the lower fractions of QRs located near micelle peripheries for samples with a high filling fraction was the increased level of aggregation in the regions between the micelles already upon casting (Figure 2c). Apparently, QR aggregation competed with QR migration toward the micelles (see Supplementary Figure S5 for even higher filling fractions). This hints that intermediate filling fractions were optimal for maximizing the fraction of QRs that organized around micelles.

The assembly behavior changed considerably when the annealing solvent was changed to chloroform. Compared to toluene, chloroform is nearly a neutral solvent for PS and P2VP (Hansen solubility parameters: δ_toluene_ = 18.2; δ_PS_ = 18.7; δ_chloroform_ = 19.0; δ_P2VP_ = 19.8) [57,58]. As the P2VP is the majority component in PS_89_-*b*-P2VP_181_, one would expect the deposited micelle cores to fuse during annealing in chloroform vapor and form continuous P2VP domains under annealing in vapors of a nonselective solvent. Figure 2g shows the onset of this process upon annealing of systems prepared with low QR fraction, where the QRs appear to have adhered to the interfaces of the reshaped P2VP domains. At higher QR fractions, the structure seemed to consist of round P2VP domains that were fused by neck-like bridges or connected through QR aggregates (Figure 2h,i; Supplementary Figure S6 confirms the attribution of dark domains to the P2VP cores). Compared to the systems annealed under toluene vapor, the QRs in the chloroform annealed systems were clearly more aggregated, consistent with the lower QR solubility in chloroform. Interestingly, it seems that the QRs, when present at sufficiently high filling fractions, helped retain the round structure of the P2VP domains that were inherited from the micellar solution. Apparently, the QR adhesion to the PS/P2VP interface, combined with their aggregation when exposed to chloroform, created physical barriers between adjacent micelles, which counteracted the tendency of the BCP to form elongated domains under the influence of the nonselective chloroform vapor.

**Figure 3:**
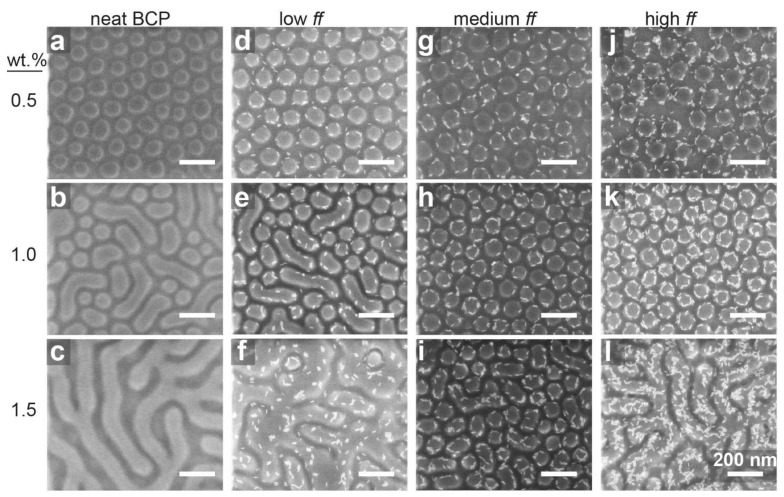
Effect of BCP concentration in nanocomposite films featuring different QR surface fractions and annealed in toluene. QR surface fractions were 0, 0.04, 0.08, and 0.13 for neat BCP (**a**–**c**), low (**d**–**f**), medium (**g**–**i**), and high (**j**–**l**) fractions, respectively.

The surfactant-like behavior of the QRs was evident when comparing the above results to the structural evolution of the deposited micellar film in the absence of QRs (Figure 3). While the micellar structure was retained in films cast from a dilute solution (0.5 wt.%) annealed in toluene vapor (Figure 3a), the P2VP domains in thick films (cast from 1.0 and 1.5 wt.% solutions) fused during annealing (Figure 3b,c). The addition of QRs at high enough concentration impeded this fusion process (Figure 3h,k). Nonetheless, for higher BCP concentrations (i.e., thicker films), the ability of QRs to retain the round shape of the P2VP domains was diminished (Figure 3f,i,l).

**Figure 4:**
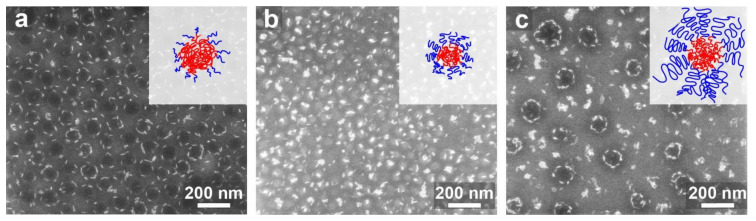
Effect of BCP composition and molecular weight in 0.5 wt.% BCP-based nanocomposites: (**a**) PS_89_-*b*-P2VP_181_ (crew-cut); (**b**) PS_102_-*b*-P2VP_97_ (symmetric); (**c**) PS_380_-*b*-P2VP_156_ (hairy). QR fractions were 0.08 in all images. The micelle cores in panel (**b**) appear light because of a charging effect.

Lastly, we wanted to see how the micelle structure affects the assembly. So far, we described the assembly with PS-*b*-P2VP featuring P2VP majority (PS_89_-*b*-P2VP_181_), which gave rise to crew-cut micelles (89 ± 9 nm core diameter) that packed densely when cast on a substrate. Using a copolymer with a similar PS block (*M*_n_ 102 kDa) and a shorter P2VP block (PS_102_-*b*-P2VP_97_) gave rise to micelles with smaller P2VP cores (38 ± 5 nm in diameter). Mixing the QRs with these micelles did not lead to circular arrangements of QRs around micelles (Figure 4b). This suggests that, when the core diameter was on the order of the length of the QR, the high curvature of the core led to a lower contact area with the QRs, which reduced the energetic gain from QR adhesion to the core surface. This conclusion is consistent with the results obtained by Sohn et al. [50] Indeed, when we used for the assembly a third type of BCP (PS_380_-*b*-P2VP_156_) that gave rise to hairy micelles (core diameter 155 ± 16 nm), circular QR arrangements were again observed (Figure 4c and Figure S7), substantiating the role of the core size in effecting this mode of organization. However, the reduced concentration of micelles left many QRs as isolated aggregates in the space between deposited micelles, especially for increased QR filling fractions.

## 4. Conclusions

This article highlighted the ability to use PS-*b*-P2VP micelles as internal templates to organize pristine quantum rods in circular arrays at the core–corona interface, without the need to chemically modify their surface. So far, only a few studies have utilized block copolymer micelles for assembling preformed nanoparticles; to our knowledge, this is the first study showing the assembly of quantum rods into an array of circular arrangements.

Monitoring the assembly of QRs into circular arrangements led to a few important insights. First, obtaining circular arrangements using micelles as templates requires the QRs to be compatible with the casting and annealing solvent and, at the same time, to be compatible with the block forming the micellar core. This requirement creates a competition for the localization preference of the QRs; in solution, they disperse in the solvent, which also solubilizes the corona-forming blocks, whereas, in the thin film, they are attracted to the core-forming blocks. Upon casting and evaporation of the solvent, the delicate balance shifts from QR–solvent toward QR–micelle interactions, leading to QR migration toward the micelles during annealing. Second, the final location of the QRs at the core–corona interfaces suggests that the QRs are not only attracted to the core-forming blocks, but also exhibit surfactant-like behavior. Indeed, it was shown that the presence of the QRs hinders micelle fusion during annealing. This behavior, however, requires a high enough concentration of QRs and low enough film thickness (i.e., corresponding to a monolayer of micelles), in which the assembly takes place in a quasi-two-dimensional fashion. Third, the micelle structure was found to play an important role on the assembly behavior of the QRs, where it was found that the core curvature must be low enough to effect circular arrangement of the QRs. Additionally, the relative length of the corona-forming blocks influences the distribution between attached QRs and QR aggregates, where crew-cut micelles shift the balance toward the circular arrangements.

The insights gained from this study open up various opportunities to use block copolymer micelles for directing the assembly of metal and semiconductor nanorods into predefined arrangements. Moreover, this approach is highly compatible with additional assembly approaches, such as the utilization of patterned substrates. These structures may lead to new ways for fabricating metasurfaces with unique optical properties (e.g., spectral operation range in the visible regime). Research in this direction is currently underway and will be reported in due course.

## Data Availability

The data presented in this study are available on request from the corresponding author.

## Supplementary Materials

The following supporting information can be downloaded at https://www.mdpi.com/article/10.3390/ma15082949/s1, Figure S1: Quantification of the changes in QR location upon annealing of 0.5 wt.% BCP micellar films containing different QR fractions (low 0.04, medium 0.08, and high 0.15). QR location is classified into four categories: QRs located at the core periphery, aggregated QRs, isolated QRs located in the region between deposited micelles, and QRs that are located on top of the micelles. Typically, 1000–2400 QRs were counted for each system; average values and standard deviations were determined from images taken at three different areas on each of the samples shown in Figure 2a–f. Figure S2: Analyses of deposited micelle dimensions of: (a,b) 0.3 wt.% neat micelles; (c,d) 0.5 wt.% neat micelles; (e,f) 0.5 wt.% QR-containing micellar film (σ_QR_ = 0.08). (a,c,e) AFM height images, representative cross-sections, and histograms generated from the images, which are deconvoluted into Gaussians reflecting the relative heights of cores and coronas (as well as QR-decorated coronas in e,f) with respect to the exposed substrate. Cyan regions demarcate the edge of the scratch that was masked for generating the height histogram; the dashed Gaussian in (b) corresponds to a region that includes a scanning artefact. (b,d,f) Representative SEM images and core diameter histograms calculated from these images. See Section 2 for additional details. Figure S3: As-cast and annealed PS (a,b) and P2VP (c,d) composite films containing QRs, cast from 1 wt.% BCP solution in toluene. Films (b,d) were annealed in toluene vapor for 5 min at room temperature. Figure S4: Nanocomposite micellar films cast from 0.5 wt.% BCP solution in toluene of QRs with different TOPO ligand densities: (a) 2.1 ligands/nm^2^, obtained after two purification cycles (as in the rest of the study); (b) 2.3 ligands/nm^2^, obtained after the first purification (centrifugation) cycle only, where the QRs appear to be adsorbed randomly to the micelles (instead of being circularly arranged). The morphology in (b) suggests that the higher ligand density makes these QRs compatible with the PS blocks (unlike the QRs in (a)), which apparently enables them to interact with the micelle coronas already in solution and, thus, end up randomly adsorbed to the micelle surfaces upon casting on the substrate. Figure S5: Nanocomposite micellar films with different QR surface fractions: (a) 0.02; (b) 0.13; (c) 0.20; (d) 0.38. Films were cast from 0.5 wt.% BCP solutions in toluene and annealed in toluene vapor. The increased level of aggregation with increasing filling fractions is evident; yet, circular QR arrangements are still visible even at the highest filling fraction (d). Figure S6: SEM images of nanocomposite micellar films (σ_QR_ = 0.10) cast from 0.5 wt.% toluene solution after annealing in chloroform vapor for (a) 15 min, and (b) 30 min. The elongation of dark domains upon extended annealing confirms their attribution to the majority (P2VP) blocks. Figure S7: SEM images of PS_380_-*b*-P2VP_156_ micellar films with different QR surface fractions cast from toluene solution at 0.5 wt.% BCP concentration: (a) σ_QR_ = 0.05; (b) σ_QR_ = 0.10 (image taken at 35° tilt).
