# Supplementary material for: Assembly of Semiconductor Nanorods into Circular Arrangements Mediated by Block Copolymer Micelles
Source: Materials (Basel). 2022 Apr 18;15(8):2949. doi: 10.3390/ma15082949 (PMC9028013; doi:10.3390/ma15082949)
Supplement: Supplementary file 1 [file materials-15-02949-s001.zip › materials-1643817-supplementary.pdf]

Article

Assembly of Semiconductor Nanorods into Circular Arrangements Mediated by Block Copolymer Micelles

Riham Muzaffar-Kawasma, Meirav Oded and Roy Shenhar *

Institute of Chemistry and the Center for Nanoscience and Nanotechnology, The Hebrew University of Jerusalem, Jerusalem 9190401, Israel; riham.kawasma@mail.huji.ac.il (R.M.-K.); meirav.oded@mail.huji.ac.il (M.O.)

* Correspondence: roys@huji.ac.il

Abstract: The collective properties of ordered ensembles of anisotropically shaped nanoparticles depend on the morphology of organization. Here we describe the utilization of block copolymer micelles to bias the natural packing tendency of semiconductor nanorods and organize them into circularly-arranged superstructures. These structures are formed as a result of a competition between the segregation tendency of the nanorods in solution and in the polymer melt: when the nanorods are highly compatible with the solvent but prefer to segregate in the melt to the core-forming block, they migrate during annealing toward the core-corona interface and their superstructure is thus templated by the shape of the micelle. The nanorods, in turn, exhibit surfactant-like behavior, and protect the micelles from coalescence during annealing. Lastly, the influence of the attributes of the micelles on nanorod organization is also studied. The circular nanorod arrangements and the insights gained in this study add to a growing list of possibilities for organizing metal and semiconducting NRs that could be achieved using rational design.

Keywords: self-assembly; block copolymer micelles; nanorods; quantum rods; nanoparticle superstructures

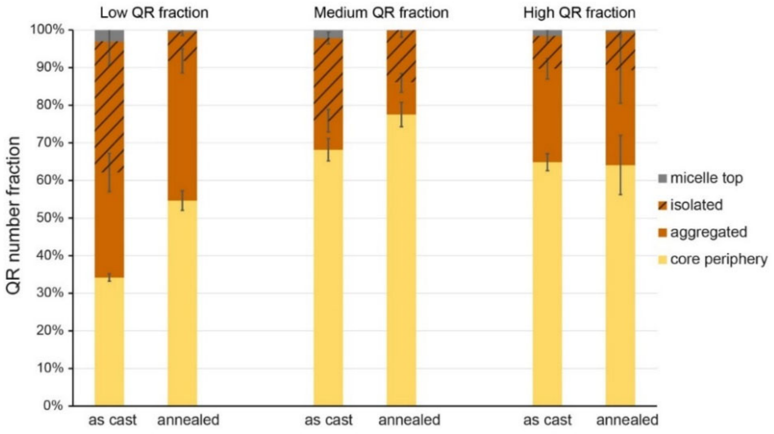

Figure S1. Quantification of the changes in QR location upon annealing of 0.5 wt.% BCP micellar films containing different QR fractions (low 0.04, medium 0.08, and high 0.15). QR location is classified into four categories: QRs located at the core periphery; aggregated QRs, isolated QRs located in the region between deposited micelles, and QRs that are located on top of the micelles. Typically 1000-2400 QRs were counted for each system; average values and standard deviations were determined from images taken at 3 different areas on each of the samples shown in Figure 2a-f.

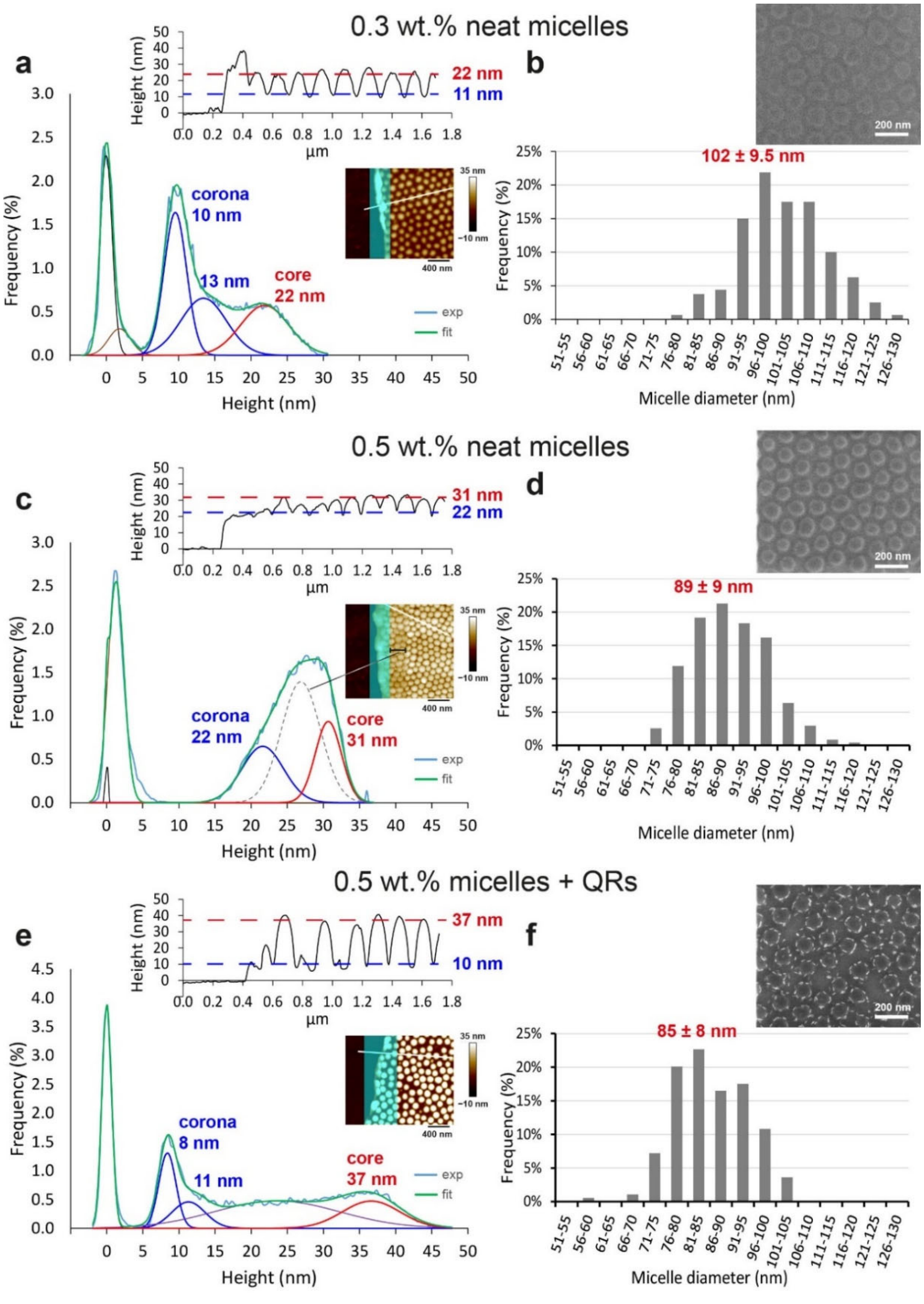

Figure S2. Analyses of deposited micelle dimensions of: (**a**,**b**) 0.3 wt.% neat micelles; (**c**,**d**) 0.5 wt.% neat micelles; (**e**,**f**) 0.5 wt.% QR-containing micellar film (σ_{QR} = 0.08). (**a**,**c**,**e**) AFM height images, representative cross-sections, and histograms generated from the the images, which are deconvoluted into Gaussians reflecting the relative heights of cores and coronas (as well as QR-decorated coronas in **e**,**f**) with respect to the exposed substrate. Cyan regions demarcate the edge of the scratch that was masked for generating the height histogram; dashed Gaussian in (**b**) correspond to a region that includes a scanning artefact. (**b**,**d**,**f**) Representative SEM images and core diameter histograms calculated from these images. See Section 2 for additional details.

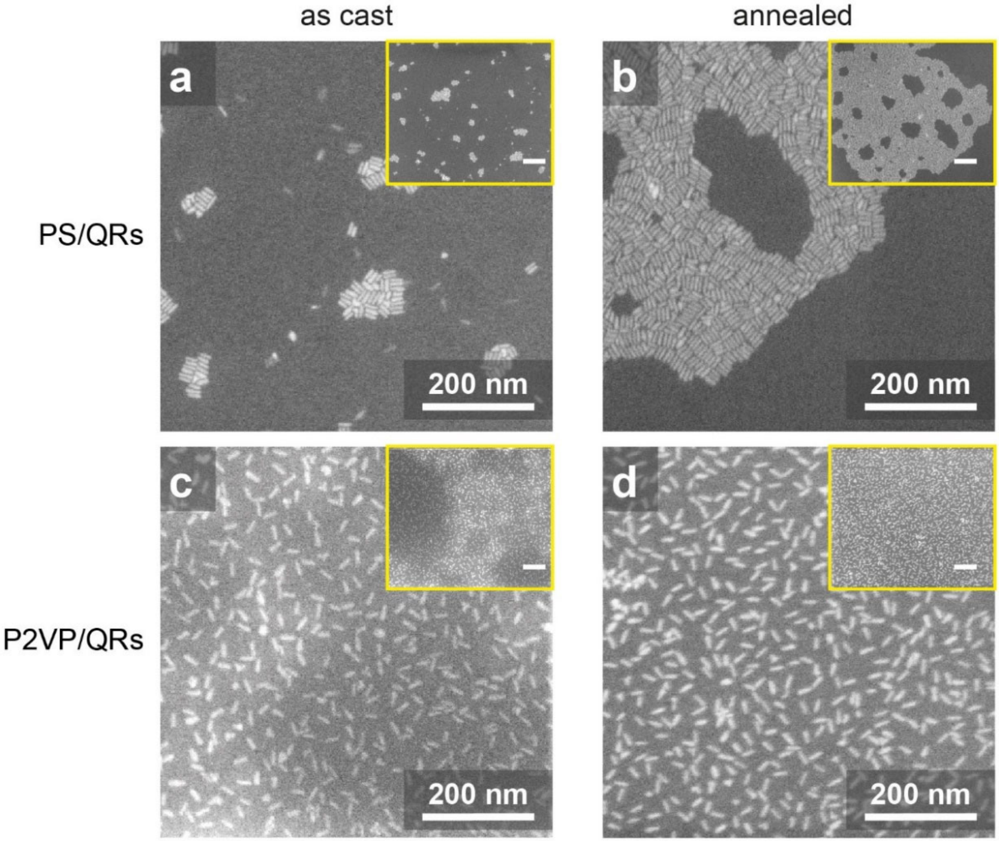

Figure S3. As-cast and annealed PS (**a**,**b**) and P2VP (**c**,**d**) composite films containing QRs, cast from 1 wt.% BCP solution in toluene. Films (**b**,**d**) were annealed in toluene vapor for 5 min at room temperature.

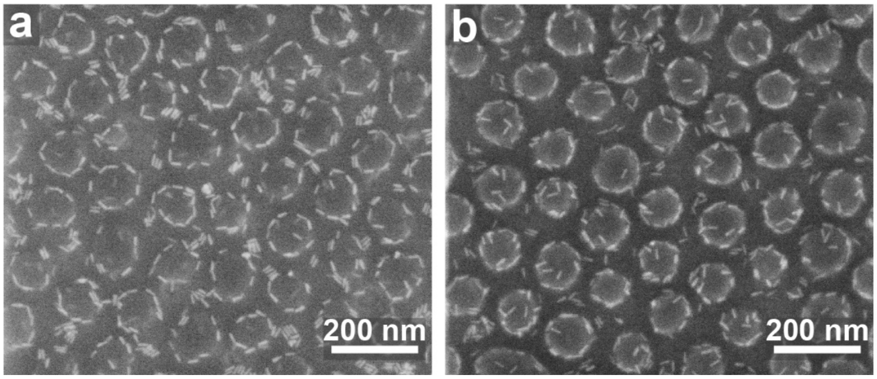

Figure S4. Nanocomposite micellar films cast from 0.5 wt.% BCP solution in toluene of QRs with different TOPO ligand densities: (**a**) 2.1 ligands/nm^2, obtained after two purification cycles (as in the rest of the study); (**b**) 2.3 ligands/nm^2, obtained after the first purification (centrifugation) cycle only, where the QRs appear to be adsorbed randomly to the micelles (instead of being circularly arranged). The morphology in (**b**) suggests that the higher ligand density makes these QRs compatible with the PS blocks (unlike the QRs in (**a**)), which apparently enables them to interact with the micelle coronas already in solution and thus end up randomly adsorbed to the micelle surfaces upon casting on the substrate.

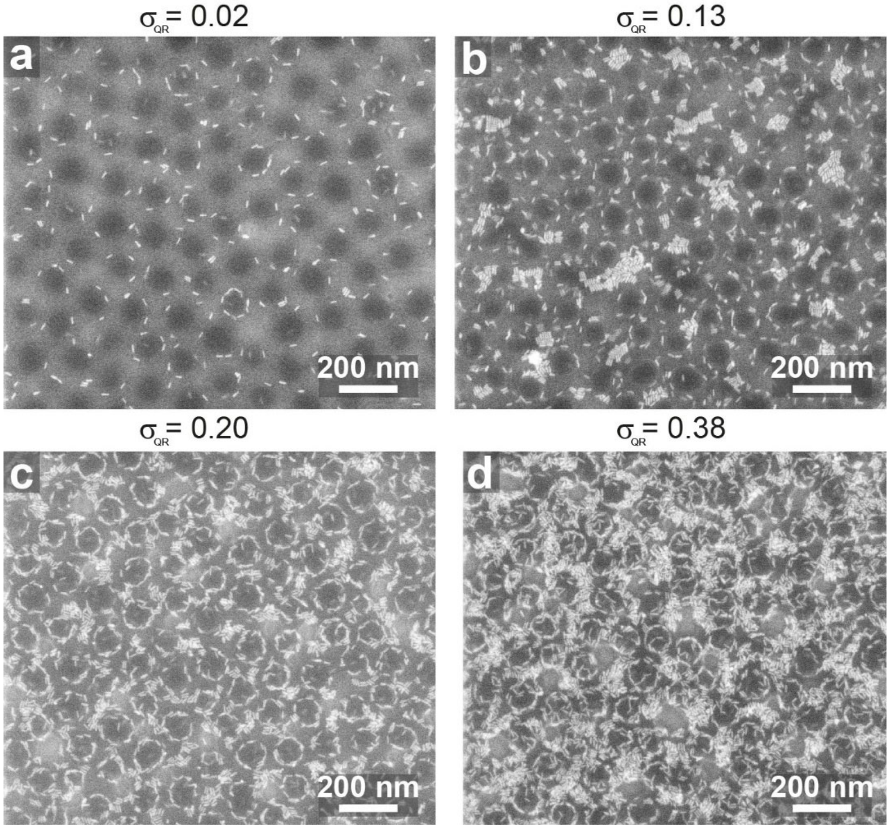

Figure S5. Nanocomposite micellar films with different QR surface fractions: (**a**) 0.02; (**b**) 0.13; (**c**) 0.20; (**d**) 0.38. Films were cast from 0.5 wt.% BCP solutions in toluene and annealed in toluene vapor. Increased level of aggregation with increasing filling fractions is evident; yet, circular QR arrangements are still visible even at the highest filling fraction (**d**).

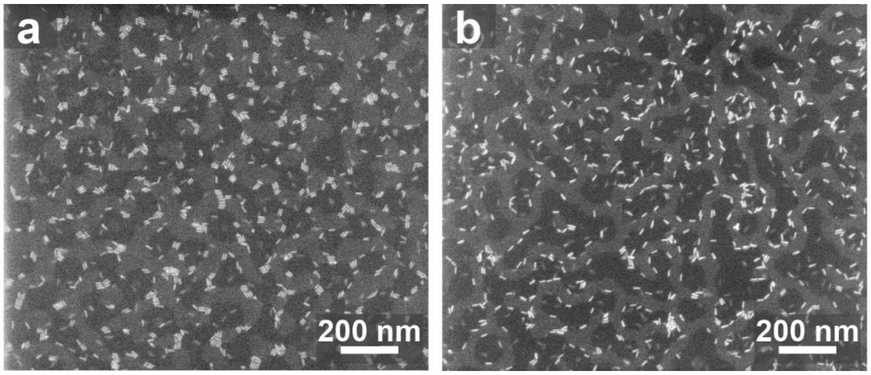

Figure S6. SEM images of nanocomposite micellar films (σ_{QR} = 0.10) cast from 0.5 wt.% toluene solution after annealing in chloroform vapor for (**a**) 15 min; (**b**) 30 min. The elongation of dark domains upon extended annealing confirms their attribution to the majority (P2VP) blocks.

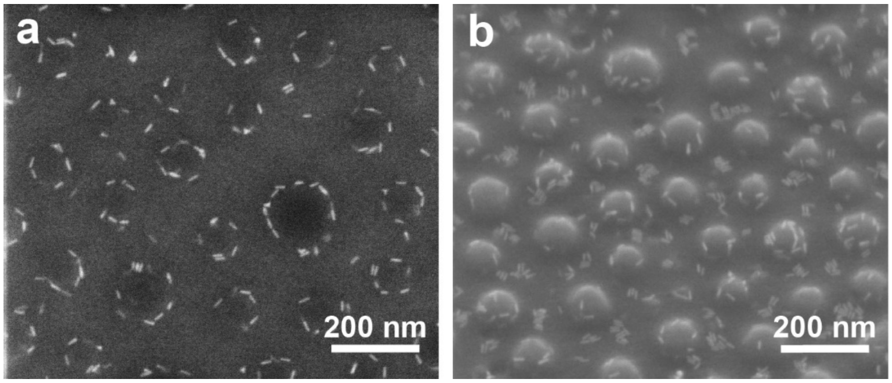

Figure S7. SEM images of PS_{380}-*b*-$P2VP_{156}$ micellar films with different QR surface fractions cast from toluene solution at 0.5 wt.% BCP concentration: (**a**) σ_{QR} = 0.05; (**b**) σ_{QR} = 0.10 (image taken at 35° tilt).
